# Harmonizing early intervention strategies: scoping review of clinical high risk for psychosis and borderline personality disorder

**DOI:** 10.3389/fpsyg.2024.1381864

**Published:** 2024-06-20

**Authors:** Gabriele Lo Buglio, Tommaso Boldrini, Andrea Polari, Flavia Fiorentino, Barnaby Nelson, Marco Solmi, Vittorio Lingiardi, Annalisa Tanzilli

**Affiliations:** ^1^Department of Dynamic and Clinical Psychology, and Health Studies, Faculty of Medicine and Psychology, Sapienza University of Rome, Rome, Italy; ^2^Department of Psychology and Educational Science, Pegaso Telematic University, Naples, Italy; ^3^Orygen Specialist Programs, Melbourne, VIC, Australia; ^4^Centre for Youth Mental Health, The University of Melbourne, Parkville, VIC, Australia; ^5^Orygen, Parkville, VIC, Australia; ^6^Department of Psychiatry, University of Ottawa, Ottawa, ON, Canada; ^7^On Track: The Champlain First Episode Psychosis Program, Department of Mental Health, The Ottawa Hospital, Ottawa, ON, Canada; ^8^Clinical Epidemiology Program, Ottawa Hospital Research Institute, University of Ottawa, Ottawa, ON, Canada; ^9^Faculty of Medicine, School of Epidemiology and Public Health, University of Ottawa, Ottawa, ON, Canada; ^10^Department of Child and Adolescent Psychiatry, Charité-Universitätsmedizin Berlin, Berlin, Germany

**Keywords:** clinical high risk for psychosis, borderline personality disorder, comorbidity, psychosis, early intervention, transdiagnostic approach, scoping review

## Abstract

**Aims:**

To map studies assessing both clinical high risk for psychosis (CHR-P) and borderline personality disorder (BPD) in clinical samples, focusing on clinical/research/preventive paradigms and proposing informed research recommendations.

**Methods:**

We conducted a PRISMA-ScR/JBI-compliant scoping review (protocol: https://osf.io/8mz7a) of primary research studies (cross-sectional/longitudinal designs) using valid measures/criteria to assess CHR-P and BPD (threshold/subthreshold) in clinical samples, reporting on CHR-P/psychotic symptoms and personality disorder(s) in the title/abstract/keywords, identified in Web of Science/PubMed/(EBSCO)PsycINFO until 23/08/2023.

**Results:**

33 studies were included and categorized into four themes reflecting their respective clinical/research/preventive paradigm: (i) BPD as a comorbidity in CHR-P youth (*k* = 20), emphasizing early detection and intervention in psychosis; (ii) attenuated psychosis syndrome (APS) as a comorbidity among BPD inpatients (*k* = 2), with a focus on hospitalized adolescents/young adults admitted for non-psychotic mental disorders; (iii) mixed samples (*k* = 7), including descriptions of early intervention services and referral pathways; (iv) transdiagnostic approaches (*k* = 4) highlighting “clinical high at risk mental state” (CHARMS) criteria to identify a pluripotent risk state for severe mental disorders.

**Conclusion:**

The scoping review reveals diverse approaches to clinical care for CHR-P and BPD, with no unified treatment strategies. Recommendations for future research should focus on: (i) exploring referral pathways across early intervention clinics to promote timely intervention; (ii) enhancing early detection strategies in innovative settings such as emergency departments; (iii) improving mental health literacy to facilitate help-seeking behaviors; (iv) analysing comorbid disorders as complex systems to better understand and target early psychopathology; (v) investigating prospective risk for BPD; (vi) developing transdiagnostic interventions; (vii) engaging youth with lived experience of comorbidity to gain insight on their subjective experience; (viii) understanding caregiver burden to craft family-focused interventions; (ix) expanding research in underrepresented regions such as Africa and Asia, and; (x) evaluating the cost-effectiveness of early interventions to determine scalability across different countries.

**Systematic Review Registration:**

https://osf.io/8mz7a.

## 1 Introduction

Adolescence and young adulthood are crucial developmental periods and, given 62.5% of mental disorders have an onset before age 25 (Solmi et al., 2022), are an important setting for the provision of early intervention strategies. These are aimed at preventing the onset of severe mental health conditions and their most adverse outcomes, including reduced life expectancy, disability, and limited academic and work attainments (Fusar-Poli et al., 2021; World Health Organization, 2022). Consistently, within the context of primary indicated prevention, early detection and intervention services have been implemented worldwide for youth manifesting the first signs and symptoms of emerging mental disorders (Shah et al., 2020).

One of the most consolidated preventive paradigms is the “clinical high-risk for psychosis” (CHR-P) paradigm, which focuses on help-seeking youth with sub-threshold psychotic symptoms, functional impairments, and presenting with up to 25% likelihood of developing a first-episode psychosis (FEP) over 3 years (Fusar-Poli et al., 2020a; Salazar de Pablo et al., 2021b). Notably, over three-quarters of CHR-P youth present with comorbid (i.e., co-existing) non-psychotic mental disorders that need clinical attention (Solmi et al., 2023). Among these, one of the most severe and potentially disabling is borderline personality disorder (BPD), which has been observed in 10% of CHR-P cases (Solmi et al., 2023) and displays a pervasive pattern of clinical manifestations, including unstable interpersonal relationships, affective instability, and self-mutilating behaviors (Chanen and Thompson, 2018; American Psychiatric Association, 2022).

Notably, BPD is also a “novel public health priority” (Chanen et al., 2017) and has been the subject of growing clinical and research interest, which has led to a specific early intervention paradigm focusing on young people with BPD and sub-syndromal borderline personality pathology (Chanen and Thompson, 2018). Clinical presentations of BPD patients are complex, and comorbid psychotic symptoms are frequently reported, with 29-50% of BPD cases experiencing auditory hallucinations (Fagioli et al., 2015; Cavelti et al., 2021).

Overall, early intervention paradigms focusing on either CHR-P or BPD show critical differences. For example, early services focusing on CHR-P (e.g., Personal Assessment and Crisis Evaluation; PACE) (Yung et al., 2007) strive to prevent the onset of full-blown psychotic disorders, whereas clinical centers focusing on BPD (e.g., Helping Young People Early; HYPE) (Chanen et al., 2009) seek to assess and address emerging severe personality disorders (PDs).

Although such services have been implemented to meet the clinical needs of different populations, CHR-P and BPD can co-exist. Moreover, they also share crucial outcomes, including high societal costs and long-term risks for self-harm, unemployment, and disability (The Public Health Group, 2005; Chanen, 2017; Fusar-Poli et al., 2020a, 2021).

However, although their co-occurrence is well-established, the consensus on the best clinical pathways for youth with both CHR-P state and BPD–even in attenuated forms–is limited, highlighting crucial shortcomings of current early paradigms. First, international clinical guidelines are specific to CHR-P (NICE, 2014; Schmidt et al., 2015) or BPD (NICE, 2009; Simonsen et al., 2019), with non-exhaustive information on the clinical management of youth with both clinical conditions. Second, treatment clinics for CHR-P and BPD may be separated and disconnected–even geographically–hindering fundamental collaborations among mental health systems and timely intervention. Third, although recent transdiagnostic approaches are promising since they “cut across,” single diagnostic entities, such models still need to be implemented at scale (Shah et al., 2020). Ironically, even though the comorbidity concept can be considered partially artifactual (Nordgaard et al., 2023), the co-existence of CHR-P and borderline personality pathology impacts “tangibly” both referral pathways of young people and decision aids of clinicians operating in mental health services. It is essential to produce research recommendations for future studies that may advance clinical care, also considering the urgent transformation for mental health argued in the recent World Health Organization (WHO) mental health report (World Health Organization, 2022).

Given this background, the current scoping review aims to explore original research on CHR-P state and BPD. This is essential to propose informed research recommendations. A scoping review design was selected (Tricco et al., 2018). In contrast with previous reviews, we do not seek to establish the meta-analytic prevalence of BPD in CHR-P samples (Boldrini et al., 2019; Solmi et al., 2023) nor explore the clinical overlap/relationship between early psychosis and BPD (West et al., 2021; Biancalani et al., 2023); instead, we aim to systematically screen and explore the body of studies including CHR-P and BPD, map clinical/research/preventive paradigms and generate informed research recommendations across preventive paradigms.

### 1.1 Review questions

(a) Which clinical/research/preventive paradigm, measures, study goals, geographic/temporal distribution, and clinical centers characterize the literature on BPD (threshold and subthreshold) and CHR-P state? (b) What are the clinical recommendations and research challenges according to the authors of relevant studies? (c) Which areas need further investigation?

## 2 Materials and methods

The proposed scoping review was performed in line with the PRISMA-ScR and JBI methodology for scoping reviews (Peters et al., 2015; Tricco et al., 2018; Peters et al., 2020; Khalil et al., 2021) and previous scoping reviews (Fornaro et al., 2021). See Supplementary material S1. The *a-priori* protocol was pre-registered in Open Science Framework (OSF: https://osf.io/8mz7a). Deviations from the original protocol are reported in the Supplementary material S2.

### 2.1 Inclusion/exclusion criteria

Included were: (a) primary research studies (i.e., “standard” research articles, letters to the Editor, brief reports, single cases, conference abstracts and, in general, “grey literature”) with any study design (e.g., randomized controlled trials, observational studies, cross-sectional and longitudinal studies), (b) focusing on clinical samples (“Population”), (c) using valid and reliable measures or diagnostic criteria to assess both BPD/BPD symptoms and CHR-P state/attenuated psychosis syndrome (APS) (“Concept”), (d) reporting information on at-risk state (or psychotic symptoms) and PDs or personality pathology (schizotypal personality disorder excluded since it is part of CHR-P inclusion criteria) in the title and/or abstract and/or keywords, and (e) written in English.

Excluded were: (a) reviews, (b) studies not focusing on clinical samples (e.g., general population), (c) not written in English. No restrictions were applied on context or geographical location (“Context”). Potential overlap among samples was not an exclusion criterion since this scoping review aimed to gather any relevant primary research study to map conceptualizations of clinical care/services, emphasizing the clinical/research “lens” adopted by the authors of each relevant study.

### 2.2 Search strategy

The search strategy aimed to identify both published and unpublished studies. A first limited search of PubMed, EBSCO/PsycINFO, and Web of Science was conducted by GLB. The initial search results were shared and discussed with the other authors of the current study. The text words contained in the titles and abstracts of relevant studies and the index terms (plus other words related to the topic of the current scoping review) were employed to develop a full search strategy for PubMed, Web of Science, and EBSCO/PsycINFO (see Supplementary material S3). The reference list of the included studies was screened for additional studies. Finally, further studies were searched on ResearchGate. A multi-step literature search was performed on Pubmed, Web of Science, and EBSCO/PsycINFO for studies published from inception to the 23rd August 2023. Citations were uploaded into Mendeley Manager/Mendeley Desktop, and duplicates were automatically excluded. GLB and a supervised student (see “Acknowledgments”) independently conducted the screening. First, titles and abstracts were checked, and then the full texts were examined. Reasons for exclusion at the full-text level were recorded. Disagreements were solved by contacting a third judge (AT).

### 2.3 Data extraction

Data were extracted by GLB. The data extracted on the characteristics of the studies was checked by FF. The following were extracted: (a) Country, sample (N, mean age, sex), type of publication (i.e., peer-review journal, grey literature, book), year, study design, and study goals; (b) Measures employed to assess BPD and CHR-P; (c) Information on other (non-borderline) PDs; (d) Research recommendations of authors of included studies; (e) Clinical recommendations of authors of included studies; (f) Concepts regarding early intervention services and early intervention strategies; (g) Potential other relevant concepts were detected, and research gaps were highlighted.

The .xls data charting file was updated while extracting the data. Potential disagreements among the authors were solved via discussion. Authors of included articles were contacted for missing or additional information.

### 2.4 Data analysis and presentation

We presented the findings in a narrative synthesis and one table and organized them into major concepts identified across the included studies. To answer the review questions (a) and (b), we organized the included studies and their data into four major concepts reflecting different clinical or research paradigms: BPD as a comorbidity among CHR-P youth (*k* = 20); attenuated psychosis syndrome (APS) as a comorbidity among BPD inpatients (*k* = 2); mixed samples (*k* = 7); transdiagnostic approaches (*k* = 4). Ten research recommendations beyond diagnostic silos were finally proposed. The results were discussed in the context of international guidelines (NICE, 2009, 2014; Schmidt et al., 2015; Simonsen et al., 2019) and the recent WHO mental health report (World Health Organization, 2022).

## 3 Results

### 3.1 Study selection

**Figure 1:**
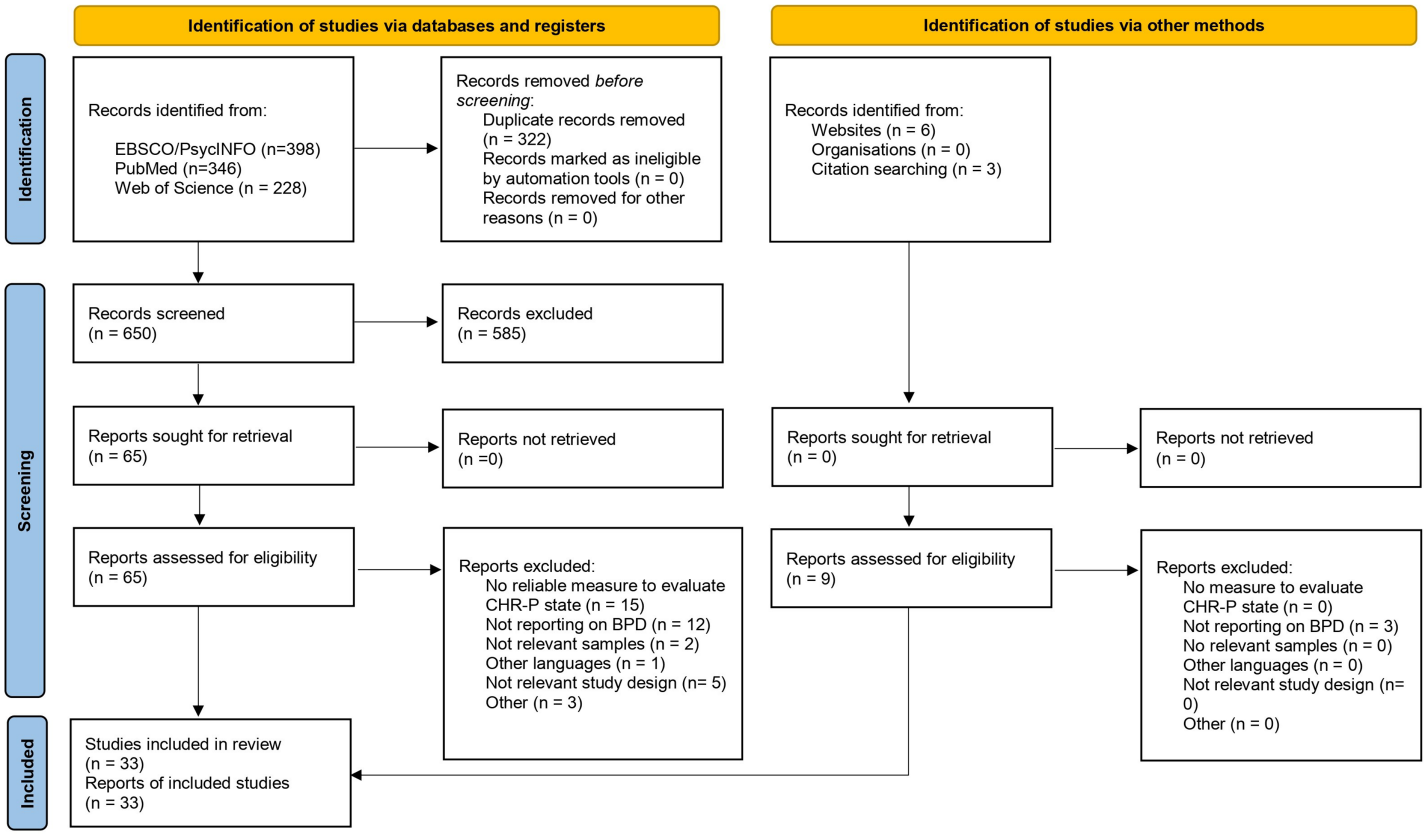
The PRISMA-ScR flow diagram of the literature search and the selection process.

972 studies were detected across registries and databases, 322 of which were duplicates, and 9 records were identified via other methods (Figure 1). 585 studies were excluded at the title-abstract level, and 41 were excluded after examining the full-texts. Reasons for exclusion at the full-text level are reported in the Supplementary material S4. We ultimately included 33 studies, and their main characteristics are displayed in Table 1. A total of 14 studies were conducted in clinical centers located in Europe, 10 in Australia, 7 in the US, and 2 studies in multiple countries. Included studies were published between 2012 and 2023, with the latter being the year with the most studies (*k* = 5). Overall, 25 publications were standard research articles, 2 were conference abstracts/conference papers, 2 were dissertations, 2 were brief reports, and 2 were Letters to the Editor. 15 studies were cross-sectional, 13 were cohort studies, and 5 were case–control studies.

**Table 1:** Characteristics of the included studies.

Authors, year	Country of the clinical service	Measures for CHR-P (or APS)	Measures for BPD	Clinical structure/service	Study population	Aims	Research type	Study design
**BPD as a comorbidity among CHR-P youth**								
Barrantes-Vidal et al. (2014)	Spain	CAARMS	SCID-II	Four mental health centers from Fundació Sant Pere Claver	35 CHR-P patients. 60% males, mean age = 20.9	To investigate childhood trauma experiences in CHR-P patients, to explore whether they differ according to gender, and to investigate their association with personality disorder traits, prodromal symptoms, and the potential moderating role of gender.	Proceeding	Cross-sectional
Boldrini et al. (2020b)	Italy	SIPS	SWAP-200-A	Child and Adolescent Neuropsychiatry Unit of the Bambino Gesù Pediatric Hospital in Rome (for recruiting CHR patients) and psychotherapy associations in Genoa, Milan, Rome, and Turin (for recruiting patients with and without PDs).	58 CHR patients, 48.3% males, mean age = 16 (SD = 1.6); 60 patients with a PD, mean age = 16 (SD = 1.6), 50% males; 59 patients without a PD, 35.6% males, mean age = 16 (SD = 1.4)	To investigate PD traits of CHR-P youth and provide a prototypic description of the most relevant personality characteristics	Standard research article	Case–control
Byars (2013)	US	SIPS	SIDP-IV	RAP Program, The Zucker Hillside Hospital, New York	150 patients, mean age = 15.5, 69% males, with several CHR-P criteria (including established ones)	To investigate the effect of personality traits on the assessment and symptom reduction of the prodrome.	Dissertation	Cohort study
Ceccolini et al. (2023)	US	SIPS	BSL–23	CEDAR, Boston	160 cis-gender patients, mean age = 17.37 (SD = 3.4) and 26 gender-expansive patients, mean Age = 18.96 (SD = 4.18)	To explore the proportion and clinical characteristics of gender-expansive patients seeking CHR-P evaluation	Standard research article	Cross-sectional
DaBreo-Otero (2021)	US	SIPS	SIDP-IV	RAP Program, The Zucker Hillside Hospital, New York	101 patients meeting different CHR-P criteria (including established ones), 70.5% males	To investigate the progression of Axis I and Axis II mental conditions. Mean follow up = 2.9 years	Dissertation	Cohort study
Fusar-Poli et al. (2017)	UK	CAARMS	ICD-10 clinical criteria	OASIS & SLaM	411 CHR-P individuals, Mean age = 23.04 (SD = 5.6), 56% males; 299 non-CHR-P individuals, Mean age = 23.21 (SD = 5.05), 57% males	To examine the long-term validity of CHR-P for predicting non-psychotic mental disorders. Mean follow-up: 1472 days (SD = 1,171 days)	Standard research article	Cohort study
Hadar et al. (2020)	Data from multiple countries	CAARMS was included	SCID-II	Ten international early psychosis clinics (including those located in Melbourne and Vienna)	304 patients, mean age: 19.12 (SD = 4.55), 46% males. 293 patients had relevant data for the study.	To explore whether BPD and SPD are more prevalent in a CHR-P sample compared to the general population; to assess whether CHR-P youth with SPD or BPD show increased rates of conversion to psychosis and more persistent attenuated psychotic symptoms than CHR-P youth without such PDs.	Letter to the Editor	Cohort study
Kotlicka-Antczak et al. (2018)	Poland	CAARMS	SCID-II	PORT programme, Central Clinical Hospital of Lodz	99 CHR-P patients, Mean age = 18.97 (3.56), 45.5% males	To characterize sociodemographic and clinical characteristics of CHR-P Polish individuals. Mean follow-up = 36.06 months (SD = 23.99)	Standard research article	Cohort study
Madsen et al. (2018)	Denmark	CAARMS	SCID-II	Psychiatric Research Facility, Copenhagen	42 CHR-P patients, 43% males, Mean age = 23.8 years (SD = 4.7).	To describe the demographics, psychopathology, and comorbid mental conditions in the first CHR-P Danish sample	Standard research article	Cross-sectional
Nelson et al. (2013)	Australia	CAARMS	SCID-II PQ-BPD	PACE, Melbourne	42 CHR-P patients, 44.9% males, Mean age = 19.22 (SD = 2.9)	Investigating whether basic self-disturbance and borderline personality pathology are associated in a CHR-P sample	Brief report	Cross-sectional
O’Connor et al. (2019)	Australia	CAARMS	DSM clinical criteria	PACE, Melbourne	59 CHR-P patients converting to psychosis, Mean age = 18.6 (SD = 2.6), 42.4% males; 59 CHR-P patients not converting to psychosis, Mean age = 18 (SD = 2.9), 40.7% males	To examine whether, at baseline entry in CHR-P clinic, perceptual abnormalities are (a) more prevalent in cases with comorbid diagnoses, (b) more prevalent in cases with childhood adversities, (c) correlated with comorbid clinical diagnoses or history of childhood adversities. Follow-up ranged from 1.2 to 6.5 years (Median = 4.5 years)	Standard research article	Cohort study
Paust et al. (2019)	Switzerland	SPI-A & SIPS	BSL-23	ZInEP, Canton Zurich	10 patients not meeting at-risk criteria, 40% of males, Mean age = 22.2 (SD = 4.89); 60 patients meeting different CHR-P criteria, 45% males, Mean age = 21.98 (SD = 5.34).	To examine borderline symptoms in patients at CHR-P and their potential impact on conversion to psychosis. Follow-up: three years	Standard research article	Cohort study
Pelizza et al. (2023)	Italy	CAARMS	DSM-IV-TR clinical criteria. Clinical assessment preferably included SCID-II	PARMS Program, Parma	52 CHR-P youth, 61.5% males (Mean age at entry = 23.42; SD = 2.97)	To describe the mental health service over the course of its clinical activity.	Standard research article	Cohort study
Rutigliano et al. (2016)	UK	CAARMS	SCID-II	OASIS, London	(a) 80 drop-out CHR-P cases (70% males), Mean age = 23.63 (SD = 4.35), (b) 74 CHR-P cases without drop-out, 50% males, Mean age = 23.20 (SD = 4.90)	To examine the impact of non-psychotic disorders on functional and clinical outcomes in a sample of CHR-P young people. Mean follow-up: 6.19 years (SD = 1.87)	Standard research article	Cohort study
Ryan et al. (2017)	Australia	CAARMS	SCID-II-PQ BPD	PACE, Melbourne	180 CHR-P patients with and without BPD (37.2% males, Mean age = 18.24, SD = 2.67 years)	To explore the type of attenuated psychotic symptoms and the prevalence of borderline personality pathology in CHR-P youth and investigate whether borderline personality pathology influences the conversion rate to psychosis. Patients underwent 6-12 months of treatment.	Standard research article	Cohort study
Schultze-Lutter et al. (2012)	Germany	SPI-A and SIPS	SAMPS	FETZ, Cologne	50 CHR-P patients who developed first-episode psychosis (males = 76%, Mean age = 24, SD = 6) and 50 CHR-P patients without conversion to psychosis (males = 76%, Mean age = 24, SD = 6)	Comparing PDs and personality accentuations, evaluated at baseline, between CHR-P patients who transitioned to psychosis and those who did not	Standard research article	Case–control
Sevilla-Llewellyn-Jones et al. (2018)	UK	CAARMS	MCMI-III	CAMEO, Cambridgeshire	40 CHR-P patients, 47.5% Males, Mean age = 21.65 (SD =2.64); 40 healthy controls, 47.5% Males, Mean age = 23 (SD = 4.79)	To investigate significant personality traits in CHR-P individuals	Standard research article	Cohort study
Thompson et al. (2012)	Australia	CAARMS	SCID-II-BPD	PACE, Melbourne	48 CHR-P patients converting to a full-blown psychotic disorder, males = 45.8%, Mean age on referral = 18.3 (SD = 2.7) and 48 CHR-P patients not converting to psychosis, males = 45.8%, mean age on referral = 18.4 (SD = 2.6)	Exploring the relationship between baseline BPD features, risk of conversion, and type of psychotic disorder developed.	Standard research article	Case–control
Tronick et al. (2023)	US	SIPS	SCID-5	Sites of the NALPS-3 study (University of North Carolina Chapel Hill, Yale University, Emory University, University of Calgary, University of California at Los Angeles, at San Diego, and at San Francisco, Harvard University, and Zucker Hillside Hospital)	684 CHR-P patients, mean age = 18.21 (SD = 4.08) and 96 healthy controls, mean age = 18.60 (SD = 4.22)	To assess the risk of violence in CHR-P patients, to identify the connection between violence risk rating scores, psychosis risk symptoms, and global functioning.	Standard research article	Cohort study
West et al. (2022)	US	SIPS	BSL-23	CEDAR, Boston	44 CHR-P individuals, 54.5% cis-male 31.8% cis-female 13.6% non-binary, Mean age = 19.4 (SD = 3.9)	To investigate BPD features with a validated self-report instrument in youth referring to a specialized CHR-P mental health center.	Brief report	Cross-sectional
**Attenuated psychotic syndrome (APS) as a comorbidity among BPD inpatients**								
Gerstenberg et al. (2015)	US	SIPS, DSM-5 criteria	SIDP-IV	Child and Adolescent Inpatient Unit of The Zucker Hillside Hospital, New York	21 APS patients, Mean age = 15 (SD = 1.4), 47.6% males; (b) 68 non-APS patients, mean age = 15.1 (SD = 1.6), 39.7% males	To evaluate the presence and characteristics of APS in a sample of hospitalized inpatients adolescents with non-psychotic disorders	Standard research article	Case–control
Salazar de Pablo et al. (2020b)	US	SIPS, DSM-5 criteria	Measures included SIDP-IV	Child and Adolescent Inpatient Unit of The Zucker Hillside Hospital, New York	Hospitalized adolescents with APS (24.6% of males, Mean = 15.5, SD = 1.3) and 183 hospitalized adolescents without APS, 32.8% of males, Mean age = 15.4 (SD = 1.5)	To characterize and compare help-seeking hospitalized adolescents with and without APS diagnosis	Standard research article	Case–control
**Mixed samples**								
Burke et al. (2022)	Australia	CAARMS	DSM-IV-TR clinical criteria (lower threshold), SCID-II-PQ BPD*	Youth Mental Health Service (Orygen) in Melbourne: EPPIC, HYPE, YMC, PACE, headspace	1,138 young people with a FEP, mean age = 19.4 (SD = 2.8). 78.6% accessed from EPPIC directly, 13.7, 3.0, 1.4%, and 3,2% patients came from PACE, HYPE, YMC, and headspace, respectively	To assess the proportion of youth attending a FEP service who had been referred via other early intervention services (i.e., ARMS, headspace, HYPE, YMC), and compare clinical and demographic characteristics and rates of admission to hospital between these cases and patients presenting directly to the FEP clinic.	Standard research article	Cross-sectional
Gajwani et al. (2022)	UK	CAARMS	SCID-II and SCID-II BPD module	NHS mental health services	(a) 30 early BPD individuals (18 subsyndromal BPD and 12 established BPD), Mean age = 19.73 (SD = 6.3), 21% males (b) 18 early psychosis individuals (12 CHR-P and 6 FEP), Mean age = 20.53 (SD = 4.3), 22% males	To investigate the clinical profiles (including adverse childhood experiences, emotional regulation difficulties, borderline personality traits, and neurodevelopmental disorders) of youth early in the course of severe mental illness	Standard research article	Cross-sectional
Gruber et al. (2023)	Austria	CAARMS, SPI-A	SCID-II	Department of Psychiatry and Psychotherapy and the Department of Psychoanalysis and Psychotherapy of the Medical University of Vienna and psychiatric departments of hospitals in Vienna and surroundings	24 CHR-P individuals, 50% males, mean age 22.55 (SD = 2.97); 29 individuals with FEP, 48.3% males, mean age 24.15 (SD = 3.70); 27 BPD individuals, males 7.4%, Mean age = 28.40 (SD = 6.49); and 27 healthy controls, males 18.5%, mean age 30.71 (SD = 11.68)	To investigate disturbances of basic self and personality functioning in FEP and CHR-P individuals compared to BPD and healthy individuals	Standard research article	Cross-sectional
Koutsouleris et al. (2014)	Data from multiple countries	ERIraos	SCID-II	Department of Psychiatry and Psychotherapy, Ludwig-Maximilian University, Munich; Hammersmith Hospital, Imperial College, London; Institute of Psychiatry, King’s College, London; Guy’s Hospital, NHS Foundation Trust, London; Washington University; Basel FePsy study	800 healthy controls, 141 individuals with schizophrenia, 104 individuals with major depression, 57 BPD individuals, and 89 CHR-P individuals. Participants were selected from a large multicenter database. The mean age ranged from 23 to 38.9 years	Explore whether patients with schizophrenia, major depression, BPD, and CHR-P deviate from the trajectory of normal brain maturation, measured as between chronological and neuroanatomical age (brain age gap estimation [BrainAGE])	Standard research article	Cross-sectional
McMillan et al. (2017)	Australia	CAARMS	DSM-IV-TR clinical criteria (lower threshold), SCID-II-PQ BPD*	Orygen Mental Health services in Melbourne: EPPIC, PACE, HYPE, YMC	103 young people, mean age = 20.9 (2.8), male cisgender 41.8%, male transgender 2.9%, female cisgender 50.5% female, transgender 0.0%, non-binary 2.9%, unsure 1.9%; N of patients recruited in the following clinics: 54 (52.4%) EPPIC, 16 (15.5 %) PACE, 20 (19.4%) HYPE, 13 (12.6%) YMC	To explore sexual functioning and subjective experience of sex of youth attending youth mental health services.	Standard research article	Cross-sectional
Sanchez et al. (2019)	Australia	CAARMS	DSM-IV-TR clinical criteria (lower threshold), SCID-II-PQ BPD*	Youth Mental Health Services in Melbourne: EPPIC, PACE, HYPE, YMC	103 youth attending the following clinics: EPPIC (54), PACE (16), HYPE (20), and YMC (13). Mean age = 20.9 (SD = 2.8), 50.5% female, 41.7% male, and 7.7% transgender	To evaluate the prevalence of high-risk sexual behaviors, sequelae, and associated factors in young patients attending specialist mental health clinics	Standard research article	Cross-sectional
Seiler et al. (2020)	Australia	CAARMS	DSM-IV-TR/DSM-5 clinical criteria (lower threshold), SCID-II-PQ BPD*	Youth Mental Health Services in Melbourne: PACE, HYPE, YMC	234 youth attending the following clinics: PACE, HYPE, YMC. 36.8% males.	To investigate the prevalence of subthreshold attenuated positive symptoms and associations between subthreshold positive symptoms and sex, migrant status, and first-degree family history of psychosis in young people attending youth mental health services	Letter to the Editor	Cross-sectional
**Transdiagnostic approaches**								
Agius et al. (2013)	UK	CAARMS	ICD clinical criteria	ASPA, Bedford	Ten adult patients, 60% males, 40% women, aged 19-26 years	To examine whether depressive symptoms corroborated the case for “Pluripotent risk syndrome” in patients previously assessed with the CAARMS.	Conference paper	Cross-sectional
Destrée et al. (2023)	Australia	CAARMS	SCID-5-PD	Headspaces and Orygen specialist program clinics in Melbourne: HYPE, YMC, PACE	43 patients, 30.23% males, mean Age = 24.02 years (SD = 2.77)	To investigate the association between obsessive-compulsive symptoms and stressful experiences while adjusting for co-occurring transdiagnostic psychiatric symptoms and distress in young adults at transdiagnostic risk	Standard research article	Cross-sectional
Hartmann et al. (2021)	Australia	CAARMS	SCID-5-PD	Headspaces and Orygen specialist program clinics in Melbourne: HYPE, YMC, PACE	68 CHARMS +, 40% males, 60% women, mean age = 19.75 (SD = 2.89); 46 CHARMS -, 35% males, 65% women, Mean age = 19.43 (SD = 4.29)	To provide a theoretical overview of clinical staging and pluripotency and to present the CHARMS approach and preliminary data of the study. Follow-up was set at 12 months.	Standard research article	Cohort study
Monego et al. (2022)	Italy	CAARMS	SCID-5	Outpatient Service for Prevention of Mental Illness, Padua University Hospital	62 help-seeking patients. 30.6% CHARMS-, 69.4% CHARMS+, Mean Age = 19.1 (SD = 2.17), 44.5% males.	To examine how functioning, depressive, and psychotic symptoms are associated with different CHARMS categories.	Standard research article	Cross-sectional

*These measures derive from Chanen et al. (2009), which provides a description of the HYPE clinic.

Population. APS: Attenuated psychosis syndrome; CHARMS: Clinical high at risk mental state; CHR-P: clinical high risk for psychosis; BPD: Borderline personality disorder; FEP: First episode of psychosis; PD: Personality disorder.

Measures. BSL-23: Borderline symptom list (Bohus et al., 2009); CAARMS: Comprehensive Assessment of At-Risk Mental States (Yung et al., 2005); DSM: Diagnostic and Statistical Manual of Mental Disorders; DSM-IV-TR: Diagnostic and Statistical Manual of Mental Disorders (4th ed., text rev.) (American Psychiatric Association, 2000); DSM-5: Diagnostic and statistical manual of mental disorders (5th ed.) (American Psychiatric Association, 2013); ERIraos: details on (Maurer et al., 2018); ICD-10: International statistical classification of diseases, 10th revision (World Health Organization, 1992); SAMPS: Selbstbeurteilung nach der Aachener Merkmalsliste für Persönlichkeitsstörungen (Woschnik and Herpertz, 1994); SCID-5: Structured Clinical Interview for DSM-5 (Osório et al., 2019); SCID-5-PD: Structured Clinical Interview for DSM-5 Personality Disorders (First et al., 2016); SCID-II: Structured Clinical Interview for DSM-5: Personality Disorders (First et al., 1997):; SCID-PQ-BPD: Structured Clinical Interview for DSM-IV (Diagnostic and Statistical Manual of Mental Disorders, Fourth Edition (American Psychiatric Association, 1994)) Axis II Personality Questionnaire borderline personality disorder items (First et al., 1997); SIPD: Structured interview for DSM-IV personality (Pfohl et al., 1997) SIPS: Structured Interview for Psychosis-Risk Syndromes (Miller et al., 2003); SPI-A: The Schizophrenia Proneness Instrument, Adult version (Schultze-Lutter et al., 2007); SWAP-200-A: Shedler–Westen Assessment Procedure-200 for Adolescents (Westen et al., 2005; DeFife et al., 2013).

Clinics/Services: ASPA: Assessment and Single point of Access team; CAMEO: Cambridgeshire and Peterborough Assessing, Managing and Enhancing Outcomes; CEDAR: Center for Early Detection, Assessment, and Response to Risk; EPPIC: Early Psychosis Prevention and Intervention Centre; FETZ: Cologne Early Recognition and Intervention Centre for mental crises; HYPE: Helping Young People Early; NAPLS: North American Prodrome Longitudinal Study; NHS: National Health Service; OASIS: Outreach and Support in South London; PORT: Programme of Recognition and Therapy; PACE: Personal Assessment and Crisis Evaluation; RAP: Recognition and Prevention Program; PARMS: Parma At-Risk Mental States; SLaM: South London and the Maudsley; NHS Foundation Trust; YMC: Youth Mood Clinic; ZInEP: Zürcher Impulsprogramm zur nachhaltigen Entwicklung in der Psychiatrie.

### 3.2 BPD as a comorbidity among CHR-P youth

20 studies (Schultze-Lutter et al., 2012; Thompson et al., 2012; Byars, 2013; Nelson et al., 2013; Barrantes-Vidal et al., 2014; Rutigliano et al., 2016; Fusar-Poli et al., 2017; Ryan et al., 2017; Kotlicka-Antczak et al., 2018; Madsen et al., 2018; Sevilla-Llewellyn-Jones et al., 2018; O’Connor et al., 2019; Paust et al., 2019; Hadar et al., 2020; Boldrini et al., 2020b; DaBreo-Otero, 2021; West et al., 2022; Ceccolini et al., 2023; Pelizza et al., 2023; Tronick et al., 2023) focused on early detection and intervention within the framework of the CHR-P paradigm. Overall, the clinical population comprised CHR-P patients and, in some studies, control patients, accessing CHR-P clinics or mental health services. CHR-P patients reported a range of comorbid mental disorders, including BPD. The studies’ goals and the clinical and research recommendations of the study authors did not focus solely on BPD, encompassing a range of clinical and research issues in the clinical management of CHR-P patients.

Specifically, clinical recommendations included evaluating at-risk mental state in samples enriched (Fusar-Poli et al., 2017), adopting clinician report measures to assess PDs (Boldrini et al., 2020b), and monitoring comorbid mental health conditions over time (Byars, 2013; Rutigliano et al., 2016; Madsen et al., 2018; Sevilla-Llewellyn-Jones et al., 2018), including BPD (Ryan et al., 2017; DaBreo-Otero, 2021) to deliver appropriate intervention (Paust et al., 2019). Other authors highlighted the role of assessing perceptual abnormalities (O’Connor et al., 2019), disturbances at different levels of selfhood (Nelson et al., 2013), and childhood trauma (Barrantes-Vidal et al., 2014; O’Connor et al., 2019) in CHR-P samples. Pelizza et al. (2023) highlighted the need to overcome the barriers between adult and child/adolescent mental health services, reduce antipsychotic dosage and delivering psychosocial interventions, and establish cultural mediation services within early intervention clinics. Other clinical recommendations included providing non-stigmatizing settings (Kotlicka-Antczak et al., 2018), fostering protective factors [e.g., social support Tronick et al., 2023], planning psychological treatments focused on underlying personality traits (Schultze-Lutter et al., 2012), and improving non-psychotic disorders and general functioning beyond preventive aims (Rutigliano et al., 2016).

Research recommendations within the CHR-P framework included developing and test new early intervention strategies for comorbid PDs, including BPD (Schultze-Lutter et al., 2012), assessing personality and/or trauma in intervention studies (Thompson et al., 2012; Hadar et al., 2020; Boldrini et al., 2020b), and investigating outcomes other than conversion to psychosis (e.g., development of non-psychotic mental disorders) (Rutigliano et al., 2016) in comparison with healthy controls (Fusar-Poli et al., 2017). West et al. (2022) emphasized research into the antecedents of symptoms. One study suggested investigating self-disturbances–for details, see (Henriksen et al., 2021)–to improve the (challenging) differential diagnosis between borderline personality pathology and schizophrenia spectrum disorders (Nelson et al., 2013; Ryan et al., 2017). Research efforts with larger samples (Sevilla-Llewellyn-Jones et al., 2018; Paust et al., 2019) and longitudinal study designs (Thompson et al., 2012; Rutigliano et al., 2016; O’Connor et al., 2019) were recommended, and the need to provide more understanding and further treatment options was emphasized (Madsen et al., 2018).

### 3.3 APS as a comorbidity among BPD inpatients

2 studies (Gerstenberg et al., 2015; Salazar de Pablo et al., 2020b) focused on patients with a wide range of mental health conditions, including BPD, with or without APS. Specifically, samples from both studies were composed of inpatient (hospitalized) adolescents or young adults admitted for non-psychotic mental disorders at the Child and Adolescent Inpatient Unit of the Zucker Hillside Hospital, New York.

Clinical recommendations in APS adolescents included age-sensitive “staged” intervention models (Gerstenberg et al., 2015). Moreover, targeting poor stress tolerance and perceptual abnormalities in need-based interventions was suggested to foster quality of life and reduce the burden experienced by both patients and their families (Salazar de Pablo et al., 2020b).

Research recommendations of Salazar De Pablo et al. (2020b) included investigating comorbid mental health conditions in APS and their relevance for the risk of developing psychosis–especially in adolescents–while Gerstenberg et al. (2015) emphasized the need for long-term prospective studies with large samples to illuminate APS and its frequency, associated characteristics, evolution from childhood to adulthood, and long-term outcomes.

### 3.4 Mixed samples

7 studies (Koutsouleris et al., 2014; McMillan et al., 2017; Sanchez et al., 2019; Seiler et al., 2020; Burke et al., 2022; Gajwani et al., 2022; Gruber et al., 2023) included patients at CHR-P and patients with BPD, with or without additional samples of patients with FEP or major depressive disorder/mood disorders and healthy controls. In this theme, CHR-P and BPD represented different clinical populations (even though some CHR-P youth also displayed a comorbid BPD). Four studies focused on Youth Mental Health Services in Melbourne, which provided descriptions of preventive services for adolescents and young adults, including HYPE (for BPD), PACE (for CHR-P), and additional early clinics for mood disorders and FEP. These studies also delivered information about referral pathways (McMillan et al., 2017; Sanchez et al., 2019; Seiler et al., 2020; Burke et al., 2022).

Clinical recommendations included assessing sub-threshold positive symptoms in help-seeking youth even though their major complaint is non-psychotic (Seiler et al., 2020), screening for neurodevelopmental disorders and adverse childhood experiences (Gajwani et al., 2022), integrating sexual health screening into initial assessment (Sanchez et al., 2019), and implementing a range of strategies to address sexual health and sexual dysfunction (McMillan et al., 2017). Gruber et al. emphasized the clinical implications of comprehensive assessment measures to evaluate identity- and self-disturbances (Gruber et al., 2023). Burke et al. (2022) argued that early intervention clinics may work alongside so-called “public health approaches”–for details, see (Ajnakina et al., 2019)–to lower the exposure to environmental factors (e.g., cannabis) associated with an increased risk for psychosis. However, other methods are needed to detect more cases at risk for psychosis. For example, youth reaching emergency departments with self-harm may be targeted by early clinics since they appear to be at increased risk for psychosis–for details, see Bolhuis (2021).

Research recommendations included employing longitudinal study designs (Gajwani et al., 2022; Gruber et al., 2023), investigating more specific neuroanatomical biomarkers (Koutsouleris et al., 2014), and replicating relevant study findings. For example, Burke et al. (2022) showed fewer voluntary and involuntary hospital admissions in youth who had transitioned to psychosis from PACE, HYPE, or primary care compared to cases presenting directly with FEP. Other authors highlighted the need for clinical pathways to address sexual health and sexual dysfunction in youth with mental health conditions (McMillan et al., 2017; Sanchez et al., 2019).

### 3.5 Transdiagnostic approaches

4 studies (Agius et al., 2013; Hartmann et al., 2021; Monego et al., 2022; Destrée et al., 2023) adopted a transdiagnostic approach, 3 of which (Hartmann et al., 2021; Monego et al., 2022; Destrée et al., 2023) applied the recent “clinical high at risk mental state” (CHARMS) criteria, which identify potentially (partially) overlapping at-risk states for psychosis, BPD, mania/bipolar disorder, and severe depressive disorder. Essential concepts are the “clinical staging” model and “pluripotency.” While the former refers to a dimensional approach that collocates the person in a continuum from an asymptomatic state to chronic and disabling conditions, the latter refers to an agnostic stance about the trajectory of mental disorders (i.e., multiple outcomes are possible) (Hartmann et al., 2021). CHARMS approach aims to capture both “homotypic progression” (e.g., an individual at CHR-P goes on to develop FEP) and “heterotypic progression” (e.g., an individual with sub-syndromal borderline personality pathology goes on to develop a major depressive disorder) (Hartmann et al., 2021).

Before developing CHARMS criteria, Agius et al. (2013) recommended using the CAARMS to assess difficult patients. Overall, an overarching goal of transdiagnostic approaches is to “maximize clinical utility” (Hartmann et al., 2021). Accordingly, research recommendations included broadening CHARMS criteria (e.g., by including also eating disorders and obsessive-compulsive disorder) (Hartmann et al., 2021), exploring conversion to different mental health conditions of each CHARMS group and their overlaps, investigating the role of transdiagnostic or specific symptoms at intake and functioning in predicting CHARMS exit mental health conditions (Monego et al., 2022), and adopting more dynamic research approaches (Hartmann et al., 2021). Finally, Destrée et al. suggested exploring the relationship between specific stressful experiences and obsessive-compulsive dimensions (Destrée et al., 2023).

## 4 Discussion

The current scoping review revealed heterogeneous clinical paradigms. Specifically, the included studies were organized into four major themes: BPD as a comorbidity among CHR-P youth, APS as a comorbidity among BPD inpatients, mixed samples, and transdiagnostic approaches. Notably, high heterogeneity was observed both across themes and within each theme. Finally, research recommendations beyond diagnostic silos were proposed.

The core finding of this scoping review is that young people with CHR-P/APS and/or BPD may be subject to a range of clinical and research paradigms. For example, BPD can be considered a comorbid mental disorder in CHR-P/APS patients that needs to be assessed and treated. Moreover, CHR-P and BPD can also represent admission diagnoses to diverse early clinics. Finally, sub-threshold psychotic symptoms and sub-threshold BPD can both be part of broader transdiagnostic approaches.

Overall, no clear therapeutic approaches have been developed for people presenting with both conditions. There is some evidence of therapeutic modalities either for BPD or CHR-P but not for both. Also, the targets of the intervention are different, with mainly transition to psychosis in CHR-P population and social and vocational functioning in BPD clinics.

Notably, the differential diagnosis is challenging since key features of a BPD diagnosis (e.g., “unstable self-image or sense of self” and experiencing “chronic feelings of emptiness”) have been consistently reported in literature focusing on schizophrenia spectrum disorders (Lingiardi, 2019; Zandersen and Parnas, 2019). This has crucial implications since patients may receive diverse treatments in highly specialized services based on diagnosis (Zandersen et al., 2019; Zandersen and Parnas, 2020).

This large body of topics and clinical and research recommendations identified in the first theme (BPD as a comorbidity among CHR-P youth) indirectly corroborates the heterogeneity of the CHR-P population observed in previous meta-research in terms of clinical presentation, clinical correlates, clinical services, and long-term outcomes (Beck et al., 2019; Fusar-Poli et al., 2020a; Catalan et al., 2021; Salazar de Pablo et al., 2021b, 2021a; Bargiota et al., 2023; Solmi et al., 2023). The second theme (APS as a comorbidity among BPD inpatients) and the fourth theme (transdiagnostic approaches) reflect a growing clinical and research interest in APS (Salazar de Pablo et al., 2020a) and transdiagnostic frameworks (Shah et al., 2020; Uhlhaas et al., 2023), respectively. Finally, some studies in the third theme (mixed samples) suggested the benefits of accessing early services before developing psychosis (e.g., reduced hospitalizations) (Burke et al., 2022), providing details into youth mental health services, entry points for potential clients, and pathways of referral to specialist clinics.

### 4.1 Research recommendations

Despite the growing body of research, early approaches are hindered by shortcomings that need to be addressed by future empirical investigations. Accordingly, we proposed 10 research recommendations (Table 2) generated by harmonizing our scoping review results with current research gaps, clinical guidelines (NICE, 2009, 2014; Schmidt et al., 2015; Simonsen et al., 2019), and the recent WHO mental health report (World Health Organization, 2022).

**Table 2:** Research recommendations.

**Transdiagnostic research recommendations**
(1) Improve research on referral pathways across early intervention services (2) Expand early detection strategies in innovative settings (e.g., emergency departments) to reduce the duration of untreated symptoms. (3) Develop programs to improve mental health literacy in the general population, improving help-seeking behaviors (4) Improve research that views BPD and CHR-P comorbidity as a complex system, adopting methods like network analysis to better understand and target early psychopathology. (5) Track BPD patients who go on to develop psychotic symptoms/track patients with sub-threshold BPD who go on to develop full-blown BPD. (6) Develop transdiagnostic interventions. (7) Engage youth with lived experience of BPD and CHR-P to gain insight into their subjective experiences for better clinical management. (8) Investigate the burden on caregivers to aid in developing interventions that support both the patient and the family system. (9) Expand research to include studies from underrepresented regions such as Asia and Africa. (10) Conduct research on the cost-effectiveness of early intervention services in various countries to assess scalability.

First, little research has focused on referral pathways of young people at risk of developing severe mental disorders. Research efforts in this field may advance coordination among different clinical services and different clinical paradigms, promoting timely intervention and appropriate referrals for each patient profile.

Second, international recommendations aim to keep the duration of untreated psychosis (i.e., the timing between the first symptom and initiation of adequate intervention) (Marshall et al., 2005) below 3 months (Bertolote and McGorry, 2005), given its prognostic significance (Howes et al., 2021). Developing early detection strategies in innovative clinical settings–e.g., emergency departments (Solmi et al., 2020)–might improve timely referral to appropriate care, reducing the duration of untreated symptoms.

Third, early clinics may be actively involved in developing programs to improve the so-called “mental health literacy” (i.e., “the ability to recognize and possess knowledge of a variety of different profiles of emerging and established mental disorders […]”) (Fusar-Poli et al., 2020b) in the general population, thus promoting help-seeking behaviors (Jorm, 2000; Jorm et al., 2006; Altuncu et al., 2023).

Fourth, there is little consensus on the best intervention for CHR-P youth with BPD (or *vice-versa*). Research efforts conceptualizing comorbid conditions as a complex system (e.g., network analysis) may improve understanding of early psychopathology manifestations and potentially suggest relevant intervention targets (Nelson et al., 2017; Borsboom et al., 2021; Ong et al., 2021; Lo Buglio et al., 2022).

Fifth, further research on the risk of developing psychosis in BPD patients may be crucial to monitor and, ideally, prevent the onset of full-blown psychotic symptoms. Moreover, further research is needed on the onset of diagnosable BPD from sub-syndromal borderline personality pathology.

Sixth, developing transdiagnostic interventions is a growing clinical and research need (Reininghaus et al., 2023).

Seventh, research engaging youth with lived experience of BPD and CHR-P may illuminate their subjective experience–for psychosis, see (Fusar-Poli et al., 2022)–promoting appropriate clinical management (Simonsen et al., 2019; Boldrini et al., 2020a; West et al., 2021).

Eight, caregivers may often need to demonstrate disabling mental health conditions in young people for whom they care to gain the attention of psychiatric services (McGorry et al., 2022). Investigating the burden experienced by caregivers may help develop comprehensive interventions considering the whole family system, further supporting the recovery process in the young person.

Ninth, none of the included studies originated from Asia and Africa, suggesting a need for research in this field across wider geographical regions.

Tenth, further cost-effectiveness research (Aceituno et al., 2019) on early intervention services in multiple countries is crucial to provide robust indications about their feasibility at scale.

### 4.2 Strengths and limitations

The main strengths of this scoping review include broad inclusion criteria, a systematic study selection process, results focusing on a range of clinical/preventive paradigms, and informed research recommendations toward paradigm integration. This study has several limitations. First, our study design did not allow for the development of clinical guidelines. Nevertheless, our study allowed for generating informed research recommendations since we harmonized findings of this scoping review with research gaps and clinical guidelines. Second, due to multiple clinical and research recommendations in the included studies, we selected and emphasized the most consistent with the aims of this current scoping review. Third, most studies were conducted in Western countries, limiting the generalizability of the findings.

## 5 Conclusion

In summary, this scoping review mapped clinical paradigms in studies on CHR-P and BPD, revealing heterogeneous conceptualizations of clinical care, preventive and research paradigms. No clear therapeutic modalities are available for people presenting with both CHR-P and BPD. Our research recommendations can be helpful to improve cooperation and knowledge integration among preventive approaches and generate evidence with real-world clinical implications.

## Data availability statement

The original contributions presented in the study are included in the article/Supplementary material, further inquiries can be directed to the corresponding author.

## Author contributions

GLB: Writing – review & editing, Writing – original draft, Project administration, Methodology, Data curation, Conceptualization. TB: Writing – review & editing, Methodology, Conceptualization. AP: Writing – review & editing, Conceptualization. FF: Writing – review & editing, Data curation. BN: Writing – review & editing. MS: Writing – review & editing, Methodology. VL: Writing – review & editing, Supervision. AT: Writing – review & editing, Supervision, Methodology, Conceptualization.
