# Supplementary material for: Harmonizing early intervention strategies: scoping review of clinical high risk for psychosis and borderline personality disorder
Source: Front Psychol. 2024 Jun 20;15:1381864. doi: 10.3389/fpsyg.2024.1381864 (PMC11223645; doi:10.3389/fpsyg.2024.1381864)
Supplement: Supplementary file 1 [file Table_1.docx]

**Supplementary materials to:**

**Title:** Harmonizing early intervention strategies: Scoping review of clinical high risk for psychosis and borderline personality disorder

**Authors:** Gabriele Lo Buglio^1^, Tommaso Boldrini^2,*^, Andrea Polari^3,4^, Flavia Fiorentino^1^, Barnaby Nelson^4,5^, Marco Solmi^6,7,8,9,10^, Vittorio Lingiardi^1,†^, & Annalisa Tanzilli^1,†^

**Affiliations:**

1. Department of Dynamic and Clinical Psychology, and Health Studies, Faculty of Medicine and Psychology, Sapienza University of Rome, Rome, Italy
2. Department of Developmental Psychology and Socialization, University of Padova, Padova, Italy
3. Orygen Specialist Programs, Melbourne, Australia
4. Centre for Youth Mental Health, The University of Melbourne, Parkville, Victoria, Australia
5. Orygen, Parkville, Victoria, Australia
6. Department of Psychiatry, University of Ottawa, Ottawa, ON, Canada
7. On Track: The Champlain First Episode Psychosis Program, Department of Mental Health, The Ottawa Hospital, Ottawa, ON, Canada.
8. Ottawa Hospital Research Institute (OHRI) Clinical Epidemiology Program, University of Ottawa, Ottawa, ON, Canada.
9. School of Epidemiology and Public Health, Faculty of Medicine, University of Ottawa, Ottawa, ON, Canada.
10. Department of Child and Adolescent Psychiatry, Charité-Universitätsmedizin Berlin, Berlin, Germany.

† These authors contributed equally to this work and share last authorship

***Correspondence:** Tommaso Boldrini, PhD, tommaso.boldrini@unipegaso.it ORCID: 0000-0003-3381-9445

**Supplementary Materials S1**

**Preferred Reporting Items for Systematic reviews and Meta-Analyses extension for Scoping Reviews (PRISMA-ScR) Checklist**

| **SECTION** | **ITEM** | **PRISMA-ScR CHECKLIST ITEM** | **REPORTED ON PAGE #** |
| --- | --- | --- | --- |
| **TITLE** | | | |
| Title | 1 | Identify the report as a scoping review. | Title page |
| **ABSTRACT** | | | |
| Structured summary | 2 | Provide a structured summary that includes (as applicable): background, objectives, eligibility criteria, sources of evidence, charting methods, results, and conclusions that relate to the review questions and objectives. | Abstract |
| **INTRODUCTION** | | | |
| Rationale | 3 | Describe the rationale for the review in the context of what is already known. Explain why the review questions/objectives lend themselves to a scoping review approach. | Y |
| Objectives | 4 | Provide an explicit statement of the questions and objectives being addressed with reference to their key elements (e.g., population or participants, concepts, and context) or other relevant key elements used to conceptualize the review questions and/or objectives. | Y |
| **METHODS** | | | |
| Protocol and registration | 5 | Indicate whether a review protocol exists; state if and where it can be accessed (e.g., a Web address); and if available, provide registration information, including the registration number. | Y |
| Eligibility criteria | 6 | Specify characteristics of the sources of evidence used as eligibility criteria (e.g., years considered, language, and publication status), and provide a rationale. | Y |
| Information sources* | 7 | Describe all information sources in the search (e.g., databases with dates of coverage and contact with authors to identify additional sources), as well as the date the most recent search was executed. | Y |
| Search | 8 | Present the full electronic search strategy for at least 1 database, including any limits used, such that it could be repeated. | Supplementary  Materials |
| Selection of sources of evidence† | 9 | State the process for selecting sources of evidence (i.e., screening and eligibility) included in the scoping review. | Y |
| Data charting process‡ | 10 | Describe the methods of charting data from the included sources of evidence (e.g., calibrated forms or forms that have been tested by the team before their use, and whether data charting was done independently or in duplicate) and any processes for obtaining and confirming data from investigators. | Y |
| Data items | 11 | List and define all variables for which data were sought and any assumptions and simplifications made. | Y |
| Critical appraisal of individual sources of evidence§ | 12 | If done, provide a rationale for conducting a critical appraisal of included sources of evidence; describe the methods used and how this information was used in any data synthesis (if appropriate). | Not applicable |
| Synthesis of results | 13 | Describe the methods of handling and summarizing the data that were charted. | Y |
| **RESULTS** | | | |
| Selection of sources of evidence | 14 | Give numbers of sources of evidence screened, assessed for eligibility, and included in the review, with reasons for exclusions at each stage, ideally using a flow diagram. | Y |
| Characteristics of sources of evidence | 15 | For each source of evidence, present characteristics for which data were charted and provide the citations. | Y; Table 1 |
| Critical appraisal within sources of evidence | 16 | If done, present data on critical appraisal of included sources of evidence (see item 12). | Not applicable |
| Results of individual sources of evidence | 17 | For each included source of evidence, present the relevant data that were charted that relate to the review questions and objectives. | Y |
| Synthesis of results | 18 | Summarize and/or present the charting results as they relate to the review questions and objectives. | Y |
| **DISCUSSION** | | | |
| Summary of evidence | 19 | Summarize the main results (including an overview of concepts, themes, and types of evidence available), link to the review questions and objectives, and consider the relevance to key groups. | Y |
| Limitations | 20 | Discuss the limitations of the scoping review process. | Y |
| Conclusions | 21 | Provide a general interpretation of the results with respect to the review questions and objectives, as well as potential implications and/or next steps. | Y |
| **FUNDING** | | | |
| Funding | 22 | Describe sources of funding for the included sources of evidence, as well as sources of funding for the scoping review. Describe the role of the funders of the scoping review. | Y |

JBI = Joanna Briggs Institute; PRISMA-ScR = Preferred Reporting Items for Systematic reviews and Meta-Analyses extension for Scoping Reviews.

* Where *sources of evidence* (see second footnote) are compiled from, such as bibliographic databases, social media platforms, and Web sites.

† A more inclusive/heterogeneous term used to account for the different types of evidence or data sources (e.g., quantitative and/or qualitative research, expert opinion, and policy documents) that may be eligible in a scoping review as opposed to only studies. This is not to be confused with *information sources* (see first footnote).

‡ The frameworks by Arksey and O’Malley (6) and Levac and colleagues (7) and the JBI guidance (4, 5) refer to the process of data extraction in a scoping review as data charting*.*

§ The process of systematically examining research evidence to assess its validity, results, and relevance before using it to inform a decision. This term is used for items 12 and 19 instead of "risk of bias" (which is more applicable to systematic reviews of interventions) to include and acknowledge the various sources of evidence that may be used in a scoping review (e.g., quantitative and/or qualitative research, expert opinion, and policy document).

*From:* Tricco AC, Lillie E, Zarin W, O'Brien KK, Colquhoun H, Levac D, et al. PRISMA Extension for Scoping Reviews (PRISMAScR): Checklist and Explanation. Ann Intern Med. 2018;169:467–473. [doi: 10.7326/M18-0850](http://annals.org/aim/fullarticle/2700389/prisma-extension-scoping-reviews-prisma-scr-checklist-explanation).

**Supplementary Materials S2**

**Minor deviations from the original protocol**

- The last search was conducted on the 23/08/2023 in order to detect recent reports;
- The possibility of the absence of comorbidity of CHR-P state and BPD was also emphasized in the abstract;
- The first research question was more specific compared to the protocol;
- We organized the study findings into four major topics detected after data extraction;
- Emphasized the potential overlap of samples among studies was not an exclusion criterion;
- We also considered the World Health Organization (WHO) mental health report (2022) with the aim to generate informed research recommendations.

**Supplementary Materials S3**

**Search strategy**

PubMed and EBSCO/PsycINFO: ((("ultra-high risk") OR ("clinical high risk") OR ("prodrom*") OR ("at-risk mental state")) AND (("psychosis") OR ("psychotic") OR ("schizophrenia")) OR ("attenuated psychotic symptoms") OR ("attenuated psychosis syndrome"))) AND (("personality disorder*") OR ("borderline personality disorder"))

Web of Science: same combination of words with one more parenthesis to run the search.

**Supplementary Materials S4**

**Excluded studies at full-text level with reasons**

| Authors and year | Reason for exclusion |
| --- | --- |
| 1. Cadenhead, 2002 | Theoretical article |
| 1. Amminger et al., 2013 | No established measure to evalute CHR-P state |
| 1. Barrantes-Vidal et al., 2013 | Not clinical sample |
| 1. Baryshnikov et al., 2018 | No established measure to evaluate CHR-P state |
| 1. Catalan et al., 2018 | No established measure to evaluate CHR-P state |
| 1. Cavelti et al., 2019 | Employing part of an established measure and not focusing on CHR-P state |
| 1. Cavelti et al., 2020 | Employing part of an established measure and not focusing on CHR-P state |
| 1. Cavelti et al., 2021 | Review |
| 1. Chanen et al., 2019 | Study protocol |
| 1. De Salve et al., 2023 | Not reporting on BPD |
| 1. Drvaric et al., 2018 | Not reporting on BPD |
| 1. Fresán et al., 2015 | Not reporting on BPD |
| 1. Hartmann et al., 2019 | The article describes the methodology of the study |
| 1. Korkeila et al., 2005 | Not reporting on BPD |
| 1. Lencz et al., 2004 | Not reporting on BPD |
| 1. Leuci et al., 2020 | Not reporting on BPD |
| 1. Lewis et al., 2000 | No established measure to evaluate CHR-P state |
| 1. Lim et al., 2018 | Not reporting on BPD |
| 1. Liu et al., 2022 | Not sure the presence of BPD-related traits |
| 1. Lundsgaard et al., 2023 | Not reporting on BPD |
| 1. Mamah et al., 2020 | Not reporting on BPD |
| 1. McDonald et al., 2021 | No established measure to evaluate CHR-P state |
| 1. Meliante et al., 2021 | Not reporting on BPD |
| 1. Morales-Munoz et al., 2020a | No established measure to evaluate CHR-P state |
| 1. Morales-Munoz et al., 2020 (Morales-Munoz et al., 2020b) | No established measure to evaluate CHR-P state |
| 1. Muntaner et al., 1988 | No established measure to evaluate CHR-P state |
| 1. Murphy et al., 2013 | No established measure to evaluate CHR-P state |
| 1. Omel’chenko et al., 2014 | Article not in English |
| 1. Rietdijk et al., 2011 | No established measure to evaluate CHR-P state |
| 1. Rodríguez Solano & González De Chávez, 2000 | No established measure to evaluate CHR-P state |
| 1. Rossi et al., 2023 | Not reporting on BPD |
| 1. Schultze-Lutter et al., 2015 | Not reporting on BPD |
| 1. Schultze-Lutter et al., 2019 | Perspective |
| 1. Sengutta et al., 2019 | No established measure to evaluate CHR-P state |
| 1. Simeonova et al., 2015 | Not reporting on BPD |
| 1. Solmi et al., 2020 | Not reporting on BPD |
| 1. Strålin and Hetta, 2021) | No established measure to evaluate CHR-P state |
| 1. Tessner et al., 2011 | Participants were recruited from the general population |
| 1. van der Gaag et al., 2019 | Not reporting on BPD |
| 1. Walker et al., 2001 | No established measure to evaluate CHR-P state |
| 1. Wang et al., 2021 | No established measure to evaluate CHR-P state |

**Supplementary Materials References**

Amminger, G.P., Chanen, A.M., Ohmann, S., Klier, C.M., Mossaheb, N., Bechdolf, A., Nelson, B., Thompson, A., McGorry, P.D., Yung Alison R. and Schaefer, M.R., 2013. Omega-3 Fatty Acid Supplementation in Adolescents With Borderline Personality Disorder and Ultra-High Risk Criteria for Psychosis: A Post Hoc Subgroup Analysis of a Double-Blind, Randomized Controlled Trial. CANADIAN JOURNAL OF PSYCHIATRY-REVUE CANADIENNE DE PSYCHIATRIE 58, 402–408. https://doi.org/10.1177/070674371305800705

Barrantes-Vidal, N., Gross, G.M., Sheinbaum, T., Mitjavila, M., Ballespí, S., Kwapil, T.R., 2013. Positive and negative schizotypy are associated with prodromal and  schizophrenia-spectrum symptoms. Schizophr Res 145, 50–55. https://doi.org/10.1016/j.schres.2013.01.007

Baryshnikov, I., Aaltonen, K., Suvisaari, J., Koivisto, M., Heikkinen, M., Joffe, G., Isometsa, E., 2018. Features of borderline personality disorder as a mediator of the relation between childhood traumatic experiences and psychosis-like experiences in patients with mood disorder. EUROPEAN PSYCHIATRY 49, 9–15. https://doi.org/10.1016/j.eurpsy.2017.12.005

Cadenhead, K.S., 2002. Vulnerability markers in the schizophrenia spectrum: implications for  phenomenology, genetics, and the identification of the schizophrenia prodrome. Psychiatr Clin North Am 25, 837–853. https://doi.org/10.1016/s0193-953x(02)00021-7

Catalan, A., de Artaza, M., Fernandez-Rivas, A., Angosto, V., Aguirregomoscorta, F., Bustamante, S., Diaz, A., Zamalloa, I., Olazabal, N., Bilbao, A., Maruottolo, C., Angel Gonzalez-Torres, M., 2018. Affectively salient signal to random noise might be used to identify psychosis vulnerability in severe mental disorders. EUROPEAN PSYCHIATRY 49, 37–42. https://doi.org/10.1016/j.eurpsy.2017.12.008

Cavelti, M., Thompson, K., Chanen, A.M., Kaess, M., 2021. Psychotic symptoms in borderline personality disorder: developmental aspects. Curr Opin Psychol 37, 26–31. https://doi.org/10.1016/j.copsyc.2020.07.003

Cavelti, M., Thompson, K., Hulbert, C., Betts, J., Jackson, H., Francey, S., Chanen, A., 2019. Preliminary evidence for the cognitive model of auditory verbal hallucinations in youth with borderline personality disorder. Front Psychiatry 10. https://doi.org/10.3389/fpsyt.2019.00292

Cavelti, M., Thompson, K., Hulbert, C., Betts, J., Jackson, H., Francey, S., McCutcheon, L., Chanen, A.M., 2020. Testing the Interpersonal-Cognitive Model of Auditory Verbal Hallucinations in Youths with Either Early-Stage Borderline Personality Disorder or First-Episode Schizophrenia Spectrum Disorder. Psychopathology 53, 23–35. https://doi.org/10.1159/000505194

Chanen, A.M., Betts, J., Jackson, H., McGorry, P., Nelson, B., Cotton, S.M., Bartholomeusz, C., Jovev, M., Ratheesh, A., Davey, C., Pantelis, C., McCutcheon, L., Francey, S., Bhaduri, A., Lowe, D., Rayner, V., Thompson, K., 2019. Aripiprazole compared with placebo for auditory verbal hallucinations in youth with borderline personality disorder: Protocol for the VERBATIM randomized controlled trial. Early Interv Psychiatry 13, 1373–1381. https://doi.org/10.1111/eip.12774

De Salve, F., Rossi, C., Cavalera, C., Lara, M., Simona, B., Sofia, T., Mauro, P., Osmano, O., 2023. Personality traits and transition to psychosis one year after the first  assessment. Front Psychol 14, 1096626. https://doi.org/10.3389/fpsyg.2023.1096626

Drvaric, L., Bagby, R.M., Kiang, M., Mizrahi, R., 2018. Maladaptive personality traits in patients identified at lower-risk and higher-risk for psychosis. Psychiatry Res 268, 348–353. https://doi.org/10.1016/j.psychres.2018.08.004

Fresán, A., León-Ortiz, P., Robles-García, R., Azcárraga, M., Guizar, D., Reyes-Madrigal, F., Tovilla-Zárate, C.A., de la Fuente-Sandoval, C., 2015. Personality features in ultra-high risk for psychosis: a comparative study with  schizophrenia and control subjects using the Temperament and Character Inventory-Revised (TCI-R). J Psychiatr Res 61, 168–173. https://doi.org/10.1016/j.jpsychires.2014.12.013

Gaag, Mark, Eurelings‐Bontekoe, L., Ising, H., Berg, David, van der Gaag, M, Eurelings-Bontekoe, L., Ising, H., van den Berg, D, Gaag, Mark, Eurelings‐Bontekoe, L., Ising, H., Berg, David, van der Gaag, M, Eurelings-Bontekoe, L., Ising, H., van den Berg, D, Gaag, Mark, Eurelings‐Bontekoe, L., Ising, H., Berg, David, van der Gaag, M, Eurelings-Bontekoe, L., Ising, H., van den Berg, D, Gaag, Mark, Eurelings‐Bontekoe, L., Ising, H., Berg, David, van der Gaag, M, Eurelings-Bontekoe, L., Ising, H., van den Berg, D, Gaag, Mark, Eurelings‐Bontekoe, L., Ising, H., Berg, David, van der Gaag, M, Eurelings-Bontekoe, L., Ising, H., van den Berg, D, Gaag, Mark, Eurelings‐Bontekoe, L., Ising, H., Berg, David, van der Gaag, M, Eurelings-Bontekoe, L., Ising, H., van den Berg, D, 2019. Ultrahigh risk for developing psychosis and psychotic personality organization. Early Interv Psychiatry 13, 673–676. https://doi.org/10.1111/eip.12687

Hartmann, J.A., Nelson, B., Spooner, R., Paul Amminger, G., Chanen, A., Davey, C.G., McHugh, M., Ratheesh, A., Treen, D., Yuen, H.P., McGorry, P.D., 2019. Broad clinical high-risk mental state (CHARMS): Methodology of a cohort study validating criteria for pluripotent risk. Early Interv Psychiatry 13, 379–386. https://doi.org/10.1111/eip.12483

Korkeila, J.A., Svirskis, T., Heinimaa, M., Ristkari, T., Huttunen, J., Ilonen, T., McGlashan, T., Salokangas, R.K.R., 2005. Substance abuse and related diagnoses in early psychosis. Compr Psychiatry 46, 447–452. https://doi.org/10.1016/j.comppsych.2005.03.008

Lencz, T., Smith, C.W., Auther, A., Correll, C.U., Cornblatt, B., 2004. Nonspecific and attenuated negative symptoms in patients at clinical high-risk  for schizophrenia. Schizophr Res 68, 37–48. https://doi.org/10.1016/S0920-9964(03)00214-7

Leuci, E., Quattrone, E., Pellegrini, P., Pelizza, L., 2020. The “Parma—Early Psychosis” program: General description and process analysis after 5 years of clinical activity. Early Interv Psychiatry 14, 356–364. https://doi.org/10.1111/eip.12897

Lewis, G., David, A.S., Malmberg, A., Allebeck, P., 2000. Non-psychotic psychiatric disorder and subsequent risk of schizophrenia. Cohort  study. Br J Psychiatry 177, 416–420. https://doi.org/10.1192/bjp.177.5.416

Lim, K.-O., Lee, T.Y., Kim, M., Chon, M.-W., Yun, J.-Y., Kim, S.N., Kwon, J.S., 2018. Early referral and comorbidity as possible causes of the declining transition rate in subjects at clinical high risk for psychosis. Early Interv Psychiatry 12, 596–604. https://doi.org/10.1111/eip.12363

Liu, C.-C., Liu, C.-M., Chien, Y.-L., Lin, Y.-T., Hsieh, M.H., Hwang, T.-J., Hwu, H.-G., 2022. Original Article Follow-up of subjects labelled with putative pre-psychotic states: Viewed from a transdiagnostic clinical high-at-risk mental state (CHARMS) paradigm. JOURNAL OF THE FORMOSAN MEDICAL ASSOCIATION 121, 1159–1166. https://doi.org/10.1016/j.jfma.2021.10.009

Lundsgaard, J., Kristensen, T.D., Nordentoft, M., Glenthøj, L.B., 2023. Premorbid functioning in adolescence associates with comorbid disorders in  individuals at ultra-high risk for psychosis: A brief report. Early Interv Psychiatry 17, 422–426. https://doi.org/10.1111/eip.13373

Mamah, D., Cloninger, C.R., Mutiso, V.N., Gitonga, I., Tele, A., Ndetei, D.M., 2020. Personality Traits as Markers of Psychosis Risk in Kenya: Assessment of  Temperament and Character. Schizophr Bull Open 1, sgaa051. https://doi.org/10.1093/schizbullopen/sgaa051

McDonald, H., Babunashvili, M., Finn, A., Willard, A., Valmaggia, L., Chadwick, P., Antonova, E., 2021. Positive schizotypy and the experience of creativity: The distinctive roles of  suspiciousness and dispositional mindfulness. Schizophr Res 228, 151–158. https://doi.org/10.1016/j.schres.2020.12.004

Meliante, M., Rossi, C., Malvini, L., Niccoli, C., Oasi, O., Barbera, S., Percudani, M., 2021. The Relationship between PID-5 Personality Traits and Mental States. A Study on a  Group of Young Adults at Risk of Psychotic Onset. Medicina (Kaunas) 57. https://doi.org/10.3390/medicina57010033

Morales-Munoz, I., Broome, M.R., Marwaha, S., 2020a. Association of Parent-Reported Sleep Problems in Early Childhood With Psychotic and Borderline Personality Disorder Symptoms in Adolescence. JAMA Psychiatry 77, 1256–1265. https://doi.org/10.1001/jamapsychiatry.2020.1875

Morales-Munoz, I., Broome, M.R., Marwaha, S., Morales-Muñoz, I., Broome, M.R., Marwaha, S., Morales-Munoz, I., Broome, M.R., Marwaha, S., 2020b. Association of Parent-Reported Sleep Problems in Early Childhood With Psychotic and Borderline Personality Disorder Symptoms in Adolescence. JAMA Psychiatry 77, 1256–1265. https://doi.org/10.1001/jamapsychiatry.2020.1875

Muntaner, C., Garcia-Sevilla, L., Fernandez, A., Torrubia, R., 1988. Personality dimensions, schizotypal and borderline personality traits and psychosis proneness. Pers Individ Dif 9, 257–268. https://doi.org/10.1016/0191-8869(88)90087-6

Murphy, J., Shevlin, M., Adamson, G., Houston, J.E., 2013. From sexual abuse to psychosis: A pilot study exploring the social deafferentation hypothesis and the mediating role of avoidance. PSYCHOSIS-PSYCHOLOGICAL SOCIAL AND INTEGRATIVE APPROACHES 5, 36–47. https://doi.org/10.1080/17522439.2011.622781

Omel’chenko, M.A., Golubev, S.A., Nikiforova, I.I.Y., Kaleda, V.G., Omelchenko, M.A., Golubev, S.A., Nikiforova, I.I.Y., Kaleda, V.G., Omel’chenko, M.A., Golubev, S.A., Nikiforova, I.I.Y., Kaleda, V.G., 2014. [Risk of manifestation of endogenous psychosis in patients with nonpsychotic  mental disorders of juvenile age]. Zh Nevrol Psikhiatr Im S S Korsakova 114, 14–20.

Rietdijk, J., Hogerzeil, S.J., van Hemert, A.M., Cuijpers, P., Linszen, D.H., van der Gaag, M., 2011. Pathways to psychosis: Help-seeking behavior in the prodromal phase. Schizophr Res 132, 213–219. https://doi.org/10.1016/j.schres.2011.08.009

Rodríguez Solano, J.J., González De Chávez, M., 2000. Premorbid personality disorders in schizophrenia. Schizophr Res 44. https://doi.org/10.1016/S0920-9964(99)00203-0

Rossi, C., De Salve, F., Biagianti, B., Cavalera, C., Malvini, L., Barbera, S., Pastelli, I., Tagliabue, S., Oasi, O., Percudani, M., 2023. At-risk mental states and personality traits: A cluster analysis approach on a  group of help-seeking young adults. Early Interv Psychiatry. https://doi.org/10.1111/eip.13397

Schultze-Lutter, F., Klosterkötter, J., Nikolaides, A., Ruhrmann, S., 2015. Personality dimensions in persons symptomatically at risk of psychosis:  pronounced but lacking a characteristic profile. Early Interv Psychiatry 9, 242–247. https://doi.org/10.1111/eip.12147

Schultze-Lutter, F., Nenadic, I., Grant, P., 2019. Psychosis and Schizophrenia-Spectrum Personality Disorders Require Early Detection on Different Symptom Dimensions. Front Psychiatry 10. https://doi.org/10.3389/fpsyt.2019.00476

Sengutta, M., Gawęda, Ł., Moritz, S., Karow, A., 2019. The mediating role of borderline personality features in the relationship between  childhood trauma and psychotic-like experiences in a sample of help-seeking non-psychotic adolescents and young adults. Eur Psychiatry 56, 84–90. https://doi.org/10.1016/j.eurpsy.2018.11.009

Simeonova, D.I., Lee, F.J., Walker, E.F., 2015. Longitudinal investigation of the relationship between family history of  psychosis and affective disorders and Child Behavior Checklist ratings in clinical high-risk adolescents. Schizophr Res 166, 24–30. https://doi.org/10.1016/j.schres.2015.04.027

Solmi, M., Campeol, M., Gentili, F., Favaro, A., Cremonese, C., 2020. Clinical presentation and need for treatment of a cohort of subjects accessing to a mental illness prevention service. Research in Psychotherapy: Psychopathology, Process and Outcome 23, 434. https://doi.org/10.4081/ripppo.2020.434

Strålin, P., Hetta, J., 2021. First episode psychosis: register-based study of comorbid psychiatric disorders  and medications before and after. Eur Arch Psychiatry Clin Neurosci 271, 303–313. https://doi.org/10.1007/s00406-020-01139-6

Tessner, K.D., Mittal, V., Walker, E.F., 2011. Longitudinal study of stressful life events and daily stressors among adolescents  at high risk for psychotic disorders. Schizophr Bull 37, 432–441. https://doi.org/10.1093/schbul/sbp087

Walker, E.F., Walder, D.J., Reynolds, F., 2001. Developmental changes in cortisol secretion in normal and at-risk youth. Dev Psychopathol 13, 721–732. https://doi.org/10.1017/S0954579401003169

Wang, Q., Zhang, L., Zhang, J., Ye, Z., Li, P., Wang, F., Cao, Y., Zhang, S., Zhou, F., Ai, Z., Zhao, N., 2021. Prevalence of Comorbid Personality Disorder in Psychotic and Non-psychotic Disorders. Front Psychiatry 12. https://doi.org/10.3389/fpsyt.2021.800047
